# Supplementary material for: A Global Screen for Assembly State Changes of the Mitotic Proteome by SEC-SWATH-MS
Source: Cell Syst. 2020 Feb 26;10(2):133–155.e6. doi: 10.1016/j.cels.2020.01.001 (PMC7042714; doi:10.1016/j.cels.2020.01.001)

Q9BY42 | RTF2_HUMAN | RTFDC1 C20orf43 AD−007 CDA05 HSPC164 HSPC169
Monomer MW [kDa]: 33.887 Monomer expected elution fraction: 50

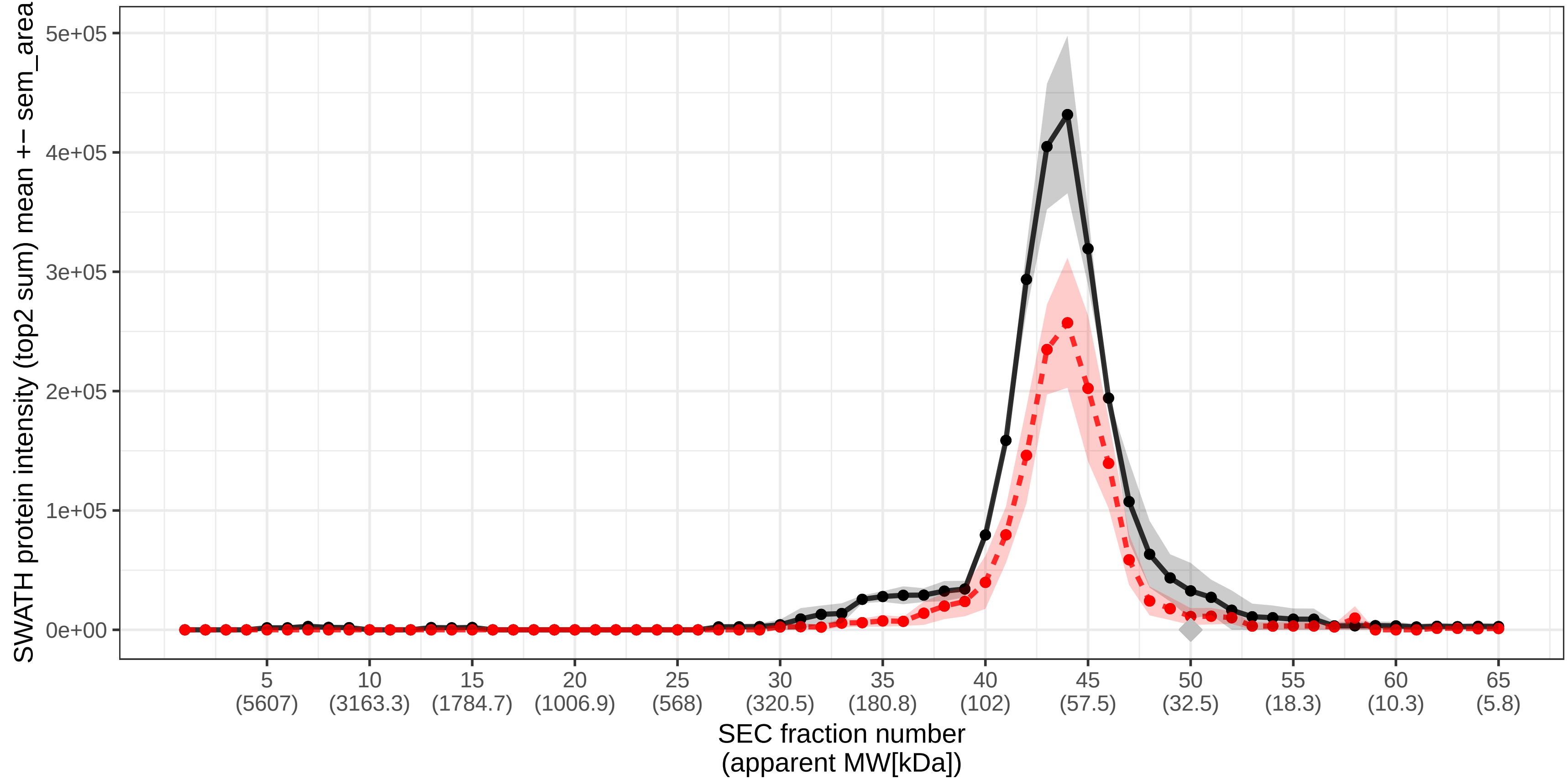

Supplement: Data S1. SEC-SWATH-MS Protein Chromatograms, Related to Figure 1 [file mmc6.zip › SECchrom_Q9BY42_RTF2_HUMAN_RTFDC1_C20orf43_AD-007_CDA05_HSP.pdf]
